# Hierarchy in materials for maximized efficiency

**DOI:** 10.1093/nsr/nwaa251

**Published:** 2020-09-26

**Authors:** Li-Hua Chen, Yu Li, Bao-Lian Su

**Affiliations:** State Key Laboratory of Advanced Technology for Materials Synthesis and Processing, Wuhan University of Technology, China; State Key Laboratory of Advanced Technology for Materials Synthesis and Processing, Wuhan University of Technology, China; State Key Laboratory of Advanced Technology for Materials Synthesis and Processing, Wuhan University of Technology, China; Laboratory of Inorganic Materials Chemistry, University of Namur, Belgium

## Abstract

This perspective article gives the future research direction on the application of the generalized Murray's law for the design of porous hierarchy in materials and the establishment of a general materials design theory 'law of hierarchy' taking four types of hierarchy into account.

## HIERARCHY

Hierarchy, originated from an ancient Greek word ‘ϵραρχα’, is a property characterized by the arrangement of a set of individual items [1] that are placed ‘above’, ‘below’ or ‘at the same level as’ one another in different systems. These items can be physical matter such as atoms, molecules, particles, objects, etc., or can be abstract and intangible entities such as names, values, categories, etc. Hierarchy, although we are not consciously aware of its existence, is an essential concept in a wide variety of fields, such as life science, materials science, computer science, philosophy, mathematics, physics, chemistry, biology, geography, topography, organizational theory, systems theory, architecture, urban construction, industrial processes and the social sciences.

Hierarchy is present everywhere in our world from nature to society, like in living systems (from simple unicellular organisms to more complex organs, such as the lung, blood circuit, kidney, trees and diatoms), ecological systems (river tributaries), and society and family organization. Every system of organization applied to the world is arranged hierarchically. Living systems and their hierarchical organizations are not only evolved and optimized by selection through hundreds of millions of years for durability, but also constructed with the capability to adapt to their external environments, to undergo self-healing, to provide protection and mobility to organisms, to help sense the environment and to perform many other highly complex functions. Owing to the characteristics of hierarchy, the living organisms realize the diffusion, the transfer, the exchange, the absorption, the storage and the transformation of substances and energy with extremely high efficiency and minimized energy consumption for survival and reproduction. The arrangement of tributaries in a river evolve to take the form of a tree's structure. Very intriguingly, such arrangement of tributaries follows a hierarchy of first, second and higher orders, with the first-order tributary being typically the smallest in size, for an optimized flow of water. Socioeconomic systems and all governments are stratified into a social hierarchy for optimized efficiency and a better governance of society. The hierarchy is thus for the optimized functions of life, ecosystem and society.

## HIERARCHY IN MATERIALS

Nature has extensively exploited the notion of hierarchy to create matter with extraordinary properties from abundant and naturally occurring building blocks. It is worth noting that the chemistry of most living organisms is restricted to elements such as carbon, nitrogen, oxygen, hydrogen, calcium, phosphorus, sulfur and silicon. This limited availability of chemical elements in the living world has forced the construction of hierarchical features as a means to enhance the overall performance of living materials. By hierarchical organizing, biological organisms are able to compensate for the shortage of chemical elements readily available in their natural environment and are able to create living materials with functionalities that would not be achievable otherwise using the same set of building blocks implemented at only one particular length scale. Hierarchy is also the characteristic of life.

Tremendous research has been focused on the development of hierarchical materials and the concept of hierarchy has been increasingly exploited in material design, synthesis and applications. However, the structure of synthetic materials remains significantly poor if compared to the sophisticated hierarchical architectures of living systems, although a large number of compounds available can be used for the construction of human-made materials. ‘Learning from nature’ has therefore been an important guiding principle in creating a new generation of synthetic hierarchical materials.

**Figure 1.**
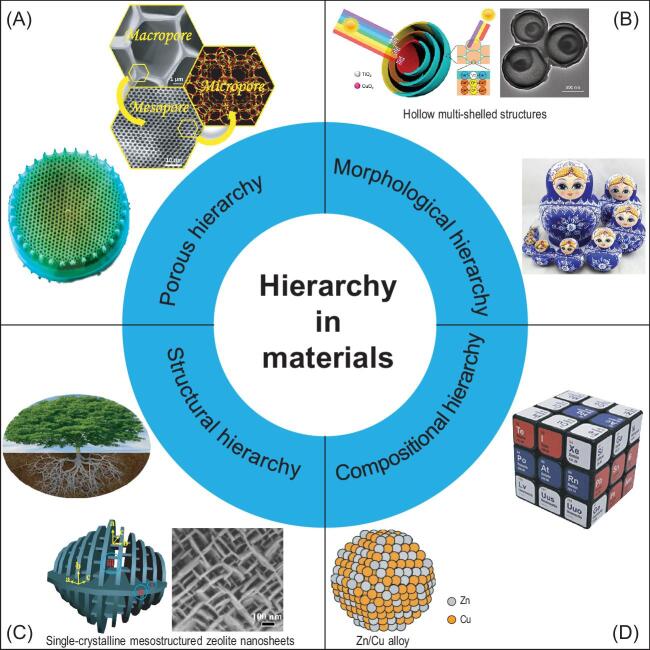
The classifications of hierarchy in materials and the representative natural and synthetic materials. (A) Porous hierarchy in diatoms. (B) Morphological hierarchy in Russian Dolls and hollow multi-shelled structures [3]. (C) Structural hierarchy in trees and single-crystalline mesostructured zeolite nanosheets [4]. (D) Compositional hierarchy in magic cubes and binary alloys [5]. Adapted with permission from refs [3–5]. Copyright 2020 Oxford Academic, 2014 Springer Nature, and 2016 American Association for the Advancement of Science.

There exist mainly four types of hierarchies in natural materials (Fig. 1): porous hierarchy, morphological hierarchy, structural hierarchy and compositional hierarchy. *Porous hierarchy:* having bimodal or multimodal porous structures with a pore size distribution from micro-, meso- to macropores in a single body enables fast mass-transport and high contact surface area for highly efficient exchange and reactions (e.g. diatom and artificial hierarchically porous zeolite single crystals [2]). *Morphological hierarchy:* having multi-level microstructure units with specific morphologies enables the collaborative coupling of functions (e.g. Russian Dolls and multi-shell hollow spheres [3]). *Structural hierarchy:* having a strict and precise repetitive combination of structural elements enables very stable construction (e.g. tree and single crystalline mesostructured zeolite nanosheets [4]). *Compositional hierarchy:* having a smart assembly of small units (including chemical composition and objects) enables the formation of a system with local and systematic variables (e.g. magic cube, human body and alloys [5]).

## POROUS HIERARCHY IN MATERIALS FOR MAXIMIZED EFFICIENCY

Introducing, simultaneously, four types of hierarchies in one material as found in nature systems constitutes a beautiful dream of scientific research. This perspective article focuses on the porous hierarchy in materials, one type of optimized structure from nature, combined with the advantages of pores at different length scales. Normally, multimodal pore structures within common composite pore materials contain pores randomly dispersed in these materials without interconnection between pores. The bimodal or multimodal porous systems can be specified as ‘hierarchical’ only if the overall pore system shows a well-ranked and interconnected porous structure and pore structure at each level has a good regularity. Thus, hierarchically porous materials contain three characteristics: *multi-levels*, *interconnectivity* and *regularity*.

Hierarchically porous architecture with a well-organized and highly interconnected pore system at different length scales is highly relevant to offering efficient mass-transfer and enhanced performance in various practical applications [6]. The micropores allow for size/shape selectivity or confinement, the mesopores for improved accessibility to active sites and mass-transfer, and the macropores for unimpeded diffusion of various molecules [7]. There has been a rapid development of synthesis strategies for tailoring hierarchical porous architecture within materials, such as zeolites, carbons, metal oxides, polymers and MOFs (metal-organic frameworks), endowing them with significantly improved performances in energy storage and conversion, catalysis, photocatalysis, adsorption, separation, gas sensing and biomedicine [7]. Generally, the synthesis approaches can be categorized into ‘*in situ*’ and ‘post-synthesis’ approaches [7]. Until now, different porosities have been successfully imbedded into porous materials, resulting in micro-mesoporosity, micro-macroporosity, meso-macroporosity, or even multimodal porosity like micro-meso-macroporosity.

## POROUS HIERARCHY IN MATERIALS: FROM EMPIRICAL SYNTHESIS TO DESIGN THEORY

Despite considerable progress in synthesis, the precise design of hierarchically porous materials with optimal properties is still in its infancy. The synthesis strategies always follow an empirical ‘trial-testing-modification-retesting’ procedure. This largely limits the advantages of hierarchically porous materials in practical applications. An integrated strategy is very much required for establishing the principle and rules among synthesis, structures (the amount, location, sizes, shapes and dimensions at different porosities) and properties. The ‘application demand-theoretical design-oriented synthesis’ should become a fundamental principle to guide the synthesis of these complex hierarchically porous materials towards their ultimately optimal functionalities. So far, scientists have theoretically designed several hierarchically porous materials with predictive and optimized performance [1,8–11]. Pérez-Ramírez *et al.* have proposed a hierarchy factor (HF) concept for the design of hierarchical zeolite catalysts [8]. The HF describes the ratio between the relative mesoporous surface area (*S*_meso_/*S*_BET_) and the relative micropore volume (*V*_micro_/*V*_pore_) of the sample. The higher the HF, the better the coordinated matching of micro- and mesoporosity, and the better catalytic performance. The HF concept considered very well the impact of microporosity and mesoporosity. Introducing the effect of macroporosity and interconnection between different pores will offer the HF concept additional power [8]. Despite these encouraging progresses in the design-synthesis concept and in the establishment of the interplay between the porous hierarchy and the enhanced performance, they still remain qualitative.

**Figure 2.**
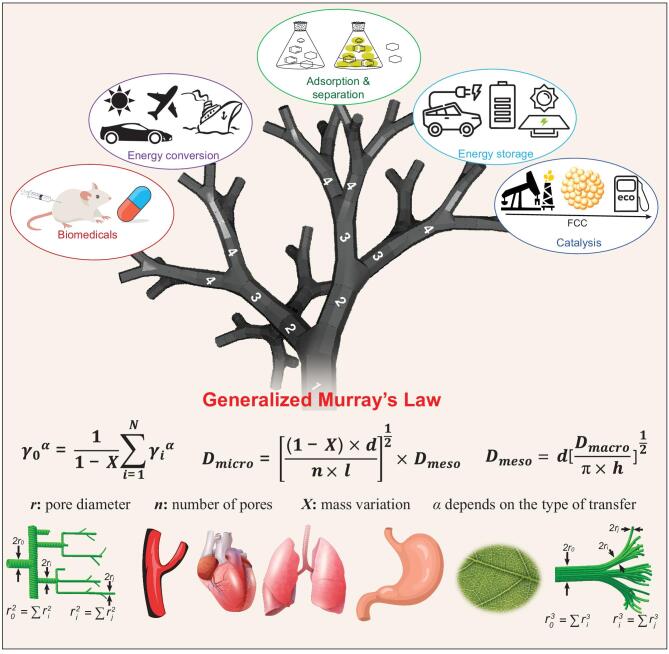
Schematic illustration of the applications of a model hierarchically porous Murray material. The equations express the generalized Murray's law and the pore size ratios between multi-scale pores established from the generalized Murray's law using microporous ZnO nanoparticles (NP) as primary building blocks to construct three-dimensional Murray material with interconnected micro-meso-macroporosity. *D*_micro_, *D*_meso_ and *D*_macro_ are the diameters of micropores, mesopores and macropores, respectively. *d* is the diameter of nanoparticles; *n* is the average number of micropores within a single NP; *h* is the thickness of material; *l* is the wall width.

Most recently, we have revealed the underlying physical principles of optimal nature systems by revisiting Murray's law, and have developed a generalized Murray's law (Equation (1)) via taking the mass variation and constant surface substance exchange into account during the mass transportation:

(1) \begin{equation*} {\gamma _0^\alpha} = \frac{1}{{1 - X}}\ \mathop \sum \limits_{i\ = \ 1}^N {\gamma _i^\alpha} , \end{equation*}

where the exponent α (2 or 3) is dependent on the type of transfer, and *X* is the ratio of mass variation during mass-transfer in the parent pores (Fig. 2) [11].

Following the generalized Murray's law, it is possible to establish the quantitative relationship between pore diameters of different length levels, and thus to guide the synthesis of bio-inspired hierarchically porous materials which respect the generalized Murray's law with maximized mass transport properties and minimized energy consumption. As an example, we emulated optimized living systems based on a bottom-up Layer-By-Layer Evaporation-Driven Self-Assembly (EDSA) approach using well-defined microporous ZnO nanoparticles as the primary building blocks, which are further assembled into three-dimensional vascularized hierarchical ZnO Murray networks with interconnected micro-meso-macropores. The relationships deduced from the generalized Murray's law gave the precise pore diameter ratios (Equations (2) and (3)):

(2) \begin{equation*} {D_{\rm micro}} = {\left[ {\frac{{\left( {1 - X} \right)\ \times d}}{{n \times l}}} \right]^{\frac{1}{2}}}\ \times {D_{\rm meso}}, \end{equation*}

 

(3) \begin{eqnarray*} {D_{\rm meso}} = d{\left[ {\frac{{{D_{\rm macro}}}}{{\pi \ \times \ h}}} \right]^{\frac{1}{2}}}. \end{eqnarray*}

Such bio-inspired Murray material has demonstrated highly enhanced mass-transfer and exchange in complex systems including liquid-solid, gas-solid and electrochemical systems [11]. This ‘learning from nature’ strategy has inspired many creative works on hierarchically porous materials, such as Murray-membranes with ultrafast water transport and evaporation for smart moisture-wicking fabrics [12], Murray-MOF film with greatly enhanced electron and mass transfer efficiency for electrochemical sensing [13] and Murray-type assembly of Co-N-C nanoparticles with efficient mass-transfer for improved electrocatalytic performance [14]. Moreover, the generalized Murray's law is also applied to construct the porous hierarchy of ZSM-5 zeolites resulting in highly enhanced catalysis performance [15].

These successful examples illustrate the power of the generalized Murray's law to guide the theoretical design of porous hierarchy in materials (Fig. 2). The application of the generalized Murray's law for the design of porous hierarchy in any kind of material will be a new important research direction of great potential and scientific value. Nevertheless, the generalized Murray's law deals only with the relationship between the pore size and the performance for optimized diffusion efficiency in materials. In addition to the effect of porous diameters, many other intrinsic characteristics of materials, such as proportion; structure; shape and surface properties of different level porosity; the number, length and angles of pore branches; the orchestrated effect of different level porosity; and the chemical composition of materials, which are also decisive for the efficiency of mass-transfer and reactions, should be introduced into the generalized Murray's law. Furthermore, in living systems, all transport, exchange and transformation of substances and energy occur at ambient conditions, the type of chemical reaction, reaction temperature and pressure all determine the final performance of the material. In this respect, improving the generalized Murray's law by taking all these parameters into consideration is a valuable strategy to optimize their properties for various applications.

Last but not least, as mentioned above, there are mainly four types of hierarchies in natural materials including porous, morphological, structural and compositional hierarchy. The establishment of a general design theory ‘law of hierarchy’, taking four types of hierarchy in materials into account, can be expected to be subversive research. With the aid of advanced artificial intelligence technologies, general principles and rules on hierarchy collected from natural examples can be converted into quantitative equations like the generalized Murray's law. The ‘application demand-theoretical design-oriented synthesis’ of hierarchically functional materials with predictive and optimized properties according to the ‘law of hierarchy’ can be achieved following ‘do as nature, work as nature and produce as nature’.
